# Interplay Between Childhood Physical Abuse and Familial Risk in the Onset of Psychotic Disorders

**DOI:** 10.1093/schbul/sbt201

**Published:** 2014-01-07

**Authors:** Helen L. Fisher, Peter McGuffin, Jane Boydell, Paul Fearon, Thomas K. Craig, Paola Dazzan, Kevin Morgan, Gillian A. Doody, Peter B. Jones, Julian Leff, Robin M. Murray, Craig Morgan

**Affiliations:** ^1^MRC Social Genetic & Developmental Psychiatry Centre, Institute of Psychiatry, King’s College London, London, UK;; ^2^Psychosis Studies, Institute of Psychiatry, King’s College London, London, UK;; ^3^Department of Psychiatry, Trinity College Dublin, Dublin, Ireland;; ^4^Health Services & Population Research, Institute of Psychiatry, King’s College London, London, UK;; ^5^National Institute of Health Research Biomedical Research Centre for Mental Health, London, UK;; ^6^Department of Psychology, University of Westminster, London, UK;; ^7^Division of Psychiatry, University of Nottingham, Nottingham, UK;; ^8^Department of Psychiatry, University of Cambridge, Cambridge, UK;; ^9^Mental Health Sciences, University College London, London, UK

**Keywords:** family history, gene-environment correlation, gene-environment interaction, liability, schizophrenia, trauma

## Abstract

*Background:* Childhood abuse is considered one of the main environmental risk factors for the development of psychotic symptoms and disorders. However, this association could be due to genetic factors influencing exposure to such risky environments or increasing sensitivity to the detrimental impact of abuse. Therefore, using a large epidemiological case-control sample, we explored the interplay between a specific form of childhood abuse and family psychiatric history (a proxy for genetic risk) in the onset of psychosis. *Methods:* Data were available on 172 first presentation psychosis cases and 246 geographically matched controls from the Aetiology and Ethnicity of Schizophrenia and Other Psychoses study. Information on childhood abuse was obtained retrospectively using the Childhood Experience of Care and Abuse Questionnaire and occurrence of psychotic and affective disorders in first degree relatives with the Family Interview for Genetic Studies. *Results:* Parental psychosis was more common among psychosis cases than unaffected controls (adjusted OR = 5.96, 95% CI: 2.09–17.01, *P* = .001). Parental psychosis was also associated with physical abuse from mothers in both cases (OR = 3.64, 95% CI: 1.06–12.51, *P* = .040) and controls (OR = 10.93, 95% CI: 1.03–115.90, *P* = .047), indicative of a gene-environment correlation. Nevertheless, adjusting for parental psychosis did not measurably impact on the abuse-psychosis association (adjusted OR = 3.31, 95% CI: 1.22–8.95, *P* **= .018). No interactions were found between familial liability and maternal physical abuse in determining psychosis caseness. *Conclusions:* This study found no evidence that familial risk accounts for associations between childhood physical abuse and psychotic disorder nor that it substantially increases the odds of psychosis among individuals reporting abuse.

## Introduction

The etiology of psychosis, and schizophrenia in particular, has been repeatedly shown to involve a major genetic component.^1^ For instance, adoption studies have reported greater prevalence of schizophrenia among individuals with an affected biological parent than those without such a parental psychiatric history.^2,3^ However, concordance rates of schizophrenia for genetically identical monozygotic twins are not 100% or even approaching it (eg, 42%),^4^ therefore indicating a role for both genetic and environmental factors in the development of the disorder.

One potential environmental risk factor is childhood abuse. Maltreatment during childhood, such as physical and sexual abuse, has been shown in prospective studies to be associated with early psychotic symptoms,^5,6^ clinically relevant psychosis,^7^ and psychotic disorders requiring treatment.^8^ In our own work, we found that exposure to childhood abuse was significantly more prevalent among first presentation psychosis patients when compared with unaffected community controls.^9^ Recent meta-analyses have confirmed that the relationship between childhood maltreatment and psychosis holds regardless of study design^10^ or type of psychotic disorder^11^ (ie, schizophrenia vs depressive psychosis). Childhood abuse thus appears to be a strong candidate for being one of the environmental risk factors involved in the etiology of psychosis.

However, it is possible that genetic factors may be confounding the abuse-psychosis relationship. A parent with psychosis may provide both a risky childhood environment and the genetic propensity for the disorder to their offspring. Indeed, having one or more biological parents with a history of psychotic disorder has been associated with a greater risk of exposure to abuse^12,13^ and also with the development of psychotic symptoms and disorders.^2,3,14,15^ Therefore, this “passive” type of gene-environment correlation (rGE)^16^ may be operating in psychosis and account for associations previously found between childhood abuse and psychotic disorders.

An individual’s genetic makeup may also influence how they react to childhood abuse and this may set in motion a chain of biological and psychological effects that lead to psychosis. This gene-environment interaction (G × E) could explain why not all individuals exposed to maltreatment go on to develop psychotic disorders,^8^ as potentially only those who also had a genetic vulnerability would be likely to become a psychosis case. Several studies have investigated interactions between genetic liability and childhood abuse in psychosis onset. However, the existing studies involving familial liability have been restricted to subclinical psychotic experiences,^5,13,17–21^ which have limited clinical utility in predicting later development of psychotic disorders,^22,23^ at least when only assessed at one timepoint.^24^ Therefore, interaction between childhood abuse and genetic vulnerability requires exploration in relation to clinically relevant psychotic disorders.

It is important to note that the G × E studies mentioned above have all used the presence of psychosis in a parent or other relative as a proxy for genetic liability. This approach may be useful given that a large number of genes, mainly of very small effect, are involved in genetic susceptibility to psychosis,^25^ rendering single candidate gene approaches extremely difficult. In essence, because individual common genetic variants each have a small main effect, detecting interactions between childhood abuse and such variants would require enormous sample sizes beyond the tens of thousands already utilized in genome-wide association studies.^26^ Family history of psychosis has the advantage of a much larger effect size but it may reflect both genetic risk and some aspects of the environment in which individuals are brought up.^27^ Additionally, as schizophrenia has a degree of genetic overlap with mood disorders,^28,29^ it seems sensible to consider interactions between childhood abuse and family history of depression and mania as well as psychosis to capture broader genetic risk for psychotic disorders. Indeed, Kramer et al^19^ recently found that having a cotwin with depression moderated the association between childhood maltreatment and psychotic-like experiences, further emphasizing the importance of utilizing an expanded familial liability factor.

Therefore, the aim of this study was to investigate the interplay between childhood abuse and family psychiatric history in the onset of psychotic disorders utilizing data from a large epidemiological study of first presentation psychosis cases and geographically matched unaffected community controls. We have previously found that severe physical abuse from mother before 12 years of age demonstrated the most robust association with psychotic disorder in this sample,^9^ and therefore in this article only rGE and G × E in relation to this type of childhood abuse are explored. Two definitions of familial risk are used: (1) a history of psychosis and (2) a history of psychosis, depression, or mania in one or more first degree relatives. We hypothesized that individuals with a parental history of psychosis or affective disorders would have a greater prevalence of both psychotic disorders and maternal physical abuse than those without this proxy genetic vulnerability. Secondly, we predicted that the association with psychotic disorders would be strongest among individuals with both exposure to maternal physical abuse and familial liability compared to those with only one or neither of these risk factors.

## Methods

### Participants

The sample was drawn from the Aetiology and Ethnicity in Schizophrenia and Other Psychoses (ÆSOP) study conducted in 1997–2000 (see Morgan et al^30^ for full details). Briefly, all patients aged 16–65 years who presented to psychiatric services for the first time with a psychotic disorder (codes F20-29 and F30-33 from the International Classification of Diseases [ICD-10]^31^) within tightly defined catchment areas in Southeast London and Nottingham were approached. Exclusion criteria included: organic psychosis; IQ under 50; previous contact with services for psychosis; and transient psychotic symptoms resulting from acute intoxication (ICD-10).^31^ Of the 469 psychosis cases identified during the study period, 390 (83%) consented to be interviewed. ICD-10 diagnoses were determined on the basis of consensus meetings involving one of ÆSOP’s principal investigators (J.L., R.M.M., P.B.J.) using data from the Schedules for Clinical Assessment in Neuropsychiatry.^32^ Diagnoses were made blind to ethnicity and abuse history.^33^

For the control group, a random sample of 391 individuals aged 16–64 years were recruited from the population of the same geographical areas as the cases. The sampling procedure was adapted from that used by the Office of Population and Census Statistics Psychiatric Morbidity Survey.^34^ To ensure that a sufficient number of people of black Caribbean ethnicity were recruited, we purposely oversampled this population by continuing recruitment for a longer period. The Psychosis Screening Questionnaire^35^ was administered to all potential control group participants; individuals were excluded if they screened positive and were found to have a psychotic disorder.

The study was approved by the South London and Maudsley NHS Trust and the Nottinghamshire NHS Trust ethics committees and all participants provided written informed consent after reading a detailed information sheet and having the opportunity to ask questions.

### Measures

Data on age, gender, ethnicity, and parental occupations during the participant’s childhood were obtained during face-to-face interviews using the Medical Research Council Sociodemographic Schedule.^36^ Ethnicity was self-ascribed and standardized using the 16 categories employed by the UK Census in 2001. The most senior occupation that participants’ fathers had held was converted into “highest ever parental social class” using the Office of National Statistics’ Socio-Economic Classification system.^37^

#### Childhood Abuse.

The Childhood Experience of Care Abuse Questionnaire (CECA.Q)^38^ was employed to retrospectively elicit information from participants concerning a range of adverse childhood experiences. For this article, only items relating to physical abuse from the main mother figure were used. This form of abuse must have commenced before age 12 to be included in the analysis. The physical abuse section begins with a screening question and a positive response is followed up with more detailed questions to ascertain the severity of abuse. Scores were dichotomized into severe and nonsevere abuse in accordance with the most conservative published cutpoints.^38^ Full details of the questionnaire are provided in Bifulco et al^38^ The CECA.Q has been shown to have good internal consistency,^39^ satisfactory levels of test-retest reliability over 7 years in this psychosis sample,^40^ and reasonable concurrent validity with existing measures.^38–40^ This questionnaire was read out to all participants to improve the accuracy of the fixed category responses obtained.

#### Familial Risk.

The Family Interview for Genetic Studies (FIGS; https://www.nimhgenetics.org/interviews/figs) was used to obtain information from a key informant (usually the mother) about the participant’s family history of mental health problems. This interview begins with a brief construction of a pedigree diagram for the participant’s first degree relatives and a series of screening questions to elicit information about possible mental health problems in these relatives. Positive responses to any of these are followed up with more specific questions to obtain symptom and treatment information for each potentially affected relative. Only 3 of these supplementary sections were chosen for this study, namely depression, mania, and psychosis. For cases, this interview was supplemented by information retrieved from clinical records. The presence or absence of a positive history in family members of an ICD-10 psychiatric diagnosis of psychosis, depression, or mania was determined through consensus meetings by 2 consultant psychiatrists utilizing the FIGS data. To maximize genetic risk, only information on first degree relatives (participant’s biological mother and father, full siblings, and children) was utilized in this article.

The FIGS consensus diagnoses were divided into several familial risk variables. Firstly, “family psychosis” denoted the presence (1) or absence (0) of a current or previous diagnosis of psychosis in at least one first degree relative. A “family mental illness” variable referred to the presence (1) or absence (0) of current or past psychosis, mania, or depression in at least one first degree relative. A “parental mental illness” variable was also created that indicated the presence (1) or absence (0) of a current or previous diagnosis of psychosis, mania, or depression in at least one biological parent. Similarly, a variable for “parental psychosis” was created that denoted the presence (1) or absence (0) of current or past psychosis in at least one biological parent.

### Analysis

All analyses were performed using Stata version 11.1 (Stata-Corp, College Station, TX). rGE was explored using binary logistic regression analysis to estimate OR of the associations between familial risk (history of parental mental illness or parental psychosis) and (1) psychotic disorder in the participants, and (2) severe physical abuse from mother before 12 years of age. The association between maternal physical abuse and psychosis was then controlled for each parental history variable to determine if genetic risk attenuated the association. Interactions between physical abuse from mother and each type of familial liability were investigated using interaction contrast ratios (ICRs)^41,42^ to estimate the relative excess risk due to interaction based on OR obtained from logistic regression analyses. This form of analysis tests for “departure from additivity” (if the odds of psychosis among individuals with both risk factors is greater than the sum of the independent effects of each risk factor). This synergistic approach is considered to be more biologically plausible for G × E than multiplicative statistical interactions^43,44^ and also aids translation of findings into clinical practice.^45^ The nlcom command in Stata was used to generate 95% CI and *P* values for the ICRs. As the numbers of cases and controls with a family history of psychosis were very small (*n* = 5 and *n* = 2, respectively), interaction analyses were only conducted for family and parental history of mental illness. Post hoc estimations of power were estimated using the “powerlog” command in Stata.

All analyses were weighted to correct for the deliberate oversampling of black Caribbean controls (see Morgan et al^46^). In the adjusted models, sex (male or female), age at interview (16–35 or 36–64 years), ethnicity (white British, white Other, black Caribbean, black African, Asian [all], or Other), study center (London or Nottingham), and highest ever parental social class (managerial/professional, intermediate, or routine/manual) were controlled.

## Results

Information on family history of mental illness was available on 172 of the 182 psychosis cases and all of the 246 controls with complete CECA.Q’s from the London and Nottingham centers of the ÆSOP study. Just over half of these cases were male (*n* = 98, 53.8%), of white British origin (*n* = 102, 56%) and from the Nottingham study center (*n* = 100, 54.9%), with an average age of 31 years (*SD* = 11.26). The cases with and without FIGS data did not differ in terms of gender (*X* ^2^ = 1.111, *P* = .345), age (*U* = 853.5, *P* = .968), or diagnosis (*X* ^2^ = 0.547, *P* = .515). The majority of the controls were female (*n* = 134, 58.1%), white British (*n* = 183, 74.4%), resided in Nottingham (*n* = 165, 67.1%), and had a mean age of 39 years (*SD* = 12.7).

In this slightly reduced sample, an almost identical association between severe maternal physical abuse before 12 years of age and psychotic disorder was found (unadjusted OR = 4.61, 95% CI: 2.00–10.63, *P* < .001; adjusted OR = 3.79, 95% CI: 1.45–9.92; *P* = .007) to that originally reported for the full sample (unadjusted OR = 4.34, 95% CI: 1.89–10.00, *P* = .001; adjusted OR = 3.60, 95% CI: 1.36–9.55, *P* = .010).^9^

### Association Between Familial Risk and Psychotic Disorder

Table 1 presents the prevalence of each type of familial liability for psychosis cases and controls along with the OR of association with case status. All types of familial risk occurred more often among psychosis cases than unaffected controls. Psychotic disorders were around 7 times more common in first degree relatives of cases than controls, while more broadly defined mental illness (psychosis, depression, or mania) was approximately 3 times more common. This indicates that familial liability should be considered as a possible explanatory variable for the previously demonstrated association between childhood abuse and psychosis. This is first explored in the context of an rGE.

**Table 1.** Prevalence of Familial Risk by Psychosis Case Status

Type of Familial Risk	Cases (*N* = 172), *n* (%)	Controls (*N* = 246), *n* (%)	Unadjusted OR^a^	95% CI	*P* Value	Adjusted OR^a,b^	95% CI	*P* Value
Family mental illness	54 (31.4)	32 (13.0)	3.24	1.95–5.37	<.001	3.92	2.25–6.83	<.001
Family psychosis	29 (17.0)	9 (3.7)	7.37	3.11–17.46	<.001	8.11	3.07–21.42	<.001
Parental mental illness	38 (22.1)	17 (6.9)	3.84	2.05–7.19	<.001	3.99	2.07–7.68	<.001
Parental psychosis	21 (12.2)	5 (2.0)	7.29	2.54–20.96	<.001	5.96	2.09–17.01	.001

*Notes*: Mental illness includes psychosis, depression, and mania.

^a^OR calculated using weighted data.

^b^Adjusted for gender, age at interview, study center, ethnicity, and highest parental social class.

### rGE for Parental Psychopathology and Maternal Physical Abuse

In order to investigate whether an rGE was operating in this sample, it was necessary to demonstrate that parental psychopathology was also associated with severe maternal physical abuse before age 12. Therefore, the reported prevalence of parental mental illness and psychosis by exposure to maternal physical abuse in childhood is presented separately for cases and controls in table 2. Parental psychopathology was more common among psychosis cases with, compared with those without, a history of maternal physical abuse. However, only associations with parental psychosis reached conventional levels of significance, with around a 3-fold increased odds of a history of psychosis in at least one parent among participants who reported abuse (*P* = .040). Even in controls, those who reported exposure to physical abuse from mother were more likely to have a parental history of psychosis than nonexposed controls. However, the extremely wide CI (1.03–115.90) indicated that this estimate was based on a very small number of controls (*n* = 9 in abused group). Comparison with a likelihood ratio test showed no evidence that the association between maternal physical abuse and parental psychosis was different for cases and controls (lrtest *X* ^2^ = 1.31, *P* = .252). The results presented in tables 1 and 2 thus suggest that an rGE is present, such that a parental history of psychosis is associated with both greater exposure to physical abuse from mother and greater odds of psychotic disorder among participants in this sample.

**Table 2.** Association Between Parental Mental Illness and Childhood Maternal Physical Abuse in Cases and Controls

Type of Parental Psychopathology	Abuse Present, *n* (%)	Abuse Absent, *n* (%)	Unadjusted OR^a^	95% CI	*P* Value	Adjusted OR^a,b^	95% CI	*P* Value
Psychosis cases	*N* = 22	*N* = 144						
Parental mental illness	6 (27.3)	30 (20.8)	1.43	0.51–3.97	.498	1.15	0.29–4.66	.840
Parental psychosis	5 (22.7)	8 (5.6)	3.64	1.06–12.51	.040	4.15	0.69–25.06	.120
Unaffected controls	*N* = 9	*N* = 229						
Parental mental illness	1 (11.1)	16 (7.0)	2.29	0.26–19.93	.453	4.29	0.33–55.35	.264
Parental psychosis	1 (11.1)	4 (1.8)	10.93	1.03–115.90	.047	4.78	0.86–26.54	.073

*Notes*: Mental illness includes psychosis, depression, and mania.

^a^OR calculated using weighted data.

^b^Adjusted for gender, age at interview, study center, ethnicity, and highest parental social class.

### Testing for Confounding by Parental Psychopathology

Given that parental psychosis was shown to be associated with both maternal physical abuse and psychosis case status, we investigated whether this form of familial risk could account for the original abuse-psychosis association, which we have previously reported.^9^ However, there was little evidence that this was the case. Thus, adjusting for a history of psychosis in at least one parent, only slightly reduced the original unadjusted OR of 4.61 (95% CI: 2.00–10.63, *P* < .001) to 3.95 (95% CI: 1.65–9.47, *P* = .002) and the adjusted OR of 3.79 (95% CI: 1.45–9.92, *P* = .007) to 3.31 (95% CI: 1.22–8.95, *P* = .018).

### Interaction Between Familial Risk and Maternal Physical Abuse

The associations between maternal physical abuse before 12 years of age and psychotic disorder are presented in table 3 stratified by family and parental mental illness along with the results of the interaction analyses. Associations were evident between maternal physical abuse and psychotic disorder regardless of whether or not participants had a family or parental history of mental illness. Though there was a trend for both risk factors to be present more often among cases than controls. However, no interactions were found between either form of familial liability and maternal physical abuse in relation to psychotic disorder in this sample. The results were largely unchanged following adjustment for potential confounders.

**Table 3.** Association Between Childhood Maternal Physical Abuse and Psychotic Disorder Among Individuals With and Without Familial Liability To Mental Illness

Type of Familial Risk	Reported Childhood Maternal Physical Abuse		Association Between Childhood Maternal Physical Abuse and Psychotic Disorder					
	Cases, *n/N* (%)	Controls, *n/N* (%)	Unadjusted OR^a^	95% CI	*P* Value	Adjusted OR^a,b^	95% CI	*P* Value
Family mental illness								
Absent	14/114 (12.3)	6/206 (2.9)	4.75	1.72–13.09	.003	3.60	1.23–10.58	.020
Present	8/52 (15.4)	3/32 (9.4)	6.86	1.67–28.24	.008	9.85	1.39–69.95	.022
			ICR: 0.24, 95% CI: −10.51 to 10.99, *P* = .965			ICR: 3.51, 95% CI: −16.16 to 23.18, *P* = .726		
Parental mental illness								
Absent	16/130 (12.3)	8/221 (3.6)	4.28	1.72–10.65	.002	3.66	1.29–10.34	.015
Present	6/36 (16.7)	1/17 (5.9)	9.49	1.13–80.00	.038	8.43	0.69–103.29	.096
			ICR: 2.91, 95% CI: −17.67 to 23.49, *P* = .782			ICR: 1.98, 95% CI: −19.48 to 23.43, *P* = .857		

*Notes*: Mental illness includes psychosis, depression, and mania. ICR, interaction contrast ratio.

^a^OR calculated using weighted data.

^b^Adjusted for gender, age at interview, study center, ethnicity, and highest parental social class.

## Discussion

Within this sample, a history of psychosis in at least one parent was around 7 times more common among participants with psychotic disorder than community controls. There was a smaller but substantial association between current or past mental illness (psychosis, depression, or mania) in a first degree relative and clinical presentation of psychosis in this sample. Associations were also found between parental history of psychosis and self-reported severe physical abuse from mother before 12 years of age. These findings together indicated the presence of an rGE. Nonetheless, controlling for parental history of psychosis only resulted in a small reduction in the strength of the association between maternal physical abuse and psychotic disorder. The second hypothesis was not supported by the findings: there was no evidence that individuals who reported exposure to childhood maternal physical abuse were more likely to have a psychotic disorder if they also had familial liability for psychotic or affective disorders compared with those without this risk factor.

### Comparisons With Previous Research

The proportion of cases reporting a first degree relative with psychosis in this sample was 17.0% which is within the range of existing studies.^47,48^ The rGE found is in keeping with previous reports of elevated rates of childhood abuse among individuals who have a parent with a psychiatric disorder.^12,13,49,50^ For instance, Walsh et al^12^ found that individuals with a parental history of psychosis, depression, or mania were 2–3 times more likely to report childhood physical, sexual, or any abuse, which is very similar to the effect size found in our study (ORs = 1.43–2.29). Although the rGE found here is likely to be of the type known as “passive,” with parents both passing on genes and creating an abusive environment, other forms of rGE could be present, eg, through the child’s genetic propensities evoking severe physical punishment.^16^ Unfortunately, it was not possible in the current study to explore such mechanisms. However, the findings of Kelleher et al^51^ indicate that such an evocative rGE is unlikely to account for associations between physical abuse and psychosis. They found that although psychotic experiences increased exposure to physical assault and other forms of victimization, reports of physical assault still predicted the development of new psychotic experiences even when this reverse causality was taken into account.

There was only a small difference in the prevalence of psychosis between those with and without a family or parental history of severe mental illness who reported exposure to maternal physical abuse. As no previous studies have explored this particular association in relation to psychotic disorders, it is not possible to make any direct comparisons with the literature. Nevertheless, Miller et al^52^ demonstrated that life events up to 25 years of age did not differ in their association with psychotic symptoms in accordance with genetic liability, and similarly Wigman et al^13^ found no interaction between parental psychosis and childhood trauma in predicting psychotic-like experiences. However, dysfunctional relationships with parents or adverse family environments in childhood have been reported to increase risk for psychosis among individuals with preexisting genetic vulnerability.^3,53–55^ These latter interactions are inconsistent with the results of the current study; the divergent findings may have been due to methodological differences, especially the focus on maternal physical abuse in this study rather than on more broadly defined forms of early adversity. Clearly, replication of the current findings is required along with greater specificity in future genetic risk studies of adverse events in childhood. Additionally, although it was not appropriate to consider other forms of childhood victimization in the current study, our findings do not preclude the possibility that other gene by victimization interactions may be occurring in psychosis and these too require exploration in future studies.

### Clinical Implications

The findings of this study have implications for the prevention of psychosis. If childhood abuse was shown to cause psychosis independently from genetic factors then eradicating abuse, or at least effectively dealing with its initial effects, would reduce the prevalence of psychotic disorders. On the other hand, if genetic factors were found to be driving the abuse-psychosis association then genes would continue to influence the development of psychosis regardless of whether abuse was prevented from occurring.^56^ In the present study, maternal physical abuse was found to be associated with psychotic disorders even when familial risk was taken into account, indicative of an independent relationship. Moreover, Kelleher et al^51^ reported that when individuals ceased to be exposed to physical abuse and other forms of victimization then their psychotic experiences reduced, and indeed the prevalence of these experiences reduced to a similar rate to that found in individuals who had never been victimized. Therefore, together these findings suggest that preventing or at least stopping continued exposure to physical abuse could prevent the onset or persistence of psychosis. Further investigation is required in clinical samples to determine if full-blown psychotic disorders could be prevented from emerging or recurring among abused individuals if exposure to abuse or other types of victimization in adolescence and adulthood were averted or individuals were better equipped to deal with the impact of (re)victimization.

### Methodological Considerations

To our knowledge, this is the first study to explore the interplay between familial liability and childhood abuse in the onset of clinically defined psychotic disorders. This study has several advantages, such as use of an epidemiologically derived sample of first presentation psychosis patients and geographically matched controls who had screened negative for psychosis, along with a standardized measure of adverse childhood experiences and inclusion of a range of demographic confounders. However, the sample size was fairly modest making it difficult to reliably detect interaction effects. Indeed, the sample was underpowered to detect associations between childhood physical abuse from mother and psychotic disorder among those with a family psychiatric history. We had only 30% power to detect the 6% difference in proportions exposed to physical abuse among individuals with a family history (*n* = 84), compared with over 90% power to detect the 9% difference in those without a family psychiatric history (*n* = 320) (see table 3). Thus, these findings require replication in larger case-control samples.

The retrospective self-report nature of assessing childhood abuse employed in this study might also render the estimated associations inaccurate. However, we have previously demonstrated in this sample that individuals with psychosis can reliably report childhood physical abuse over time, across measures, and regardless of symptom severity and content.^40^ Additionally, we were not able to control for the potential impact of cannabis use within these analyses and this may have resulted in overestimations of the main effects as it has previously been associated with both childhood physical abuse^57^ and parental psychopathology.^58^

The separation of family history data into a dichotomous variable has been criticized for being an inadequate reflection of familial liability^59^ and more comprehensive scores could be obtained by considering the number of affected relatives and passage through the age of risk for unaffected relatives. Unfortunately, it was not possible in the current study to calculate a more sensitive measure of familial genetic risk as the information on the pedigree diagrams was often limited especially regarding the age of siblings and children. Therefore, future cases of psychosis among the younger relatives may have been missed and estimations of the degree of genetic loading for disorder could not be made. Consequently, the impact of familial genetic risk in this sample might have been underestimated. Nonetheless, Milne et al^60^ tested several methods of calculating family psychiatric history and concluded that, given the extremely low prevalence of psychotic disorders, a simple present/absent dichotomous measure of having one or more first degree relatives with psychosis was satisfactory for these disorders.

A further potential limitation is the use of the psychiatric status of biological parents and other first degree relatives as a proxy for participants’ genotype. This is not a particularly sensitive method, and has been criticized on the basis that offspring share only half of their parents’ genetic material and developmental effects may dilute this shared inheritance further. As Gottesman and Bertelsen^61^ highlighted, it is also possible that parents pass on a genetic vulnerability to psychosis without overtly manifesting the disorder themselves (the phenotype is not expressed). Additionally, there is often a lack of correspondence between parental psychopathology and the type of disorder their offspring develop.^62^ Moreover, genetic risk factors may not necessarily be passed on by affected family members instead they may occur through spontaneous mutations^63^ or be environmentally mediated.^64^ Subsequently, in order to address this issue comprehensively, specific genes and their polymorphisms need to be investigated, ideally in extremely large samples so that several genes (and environments) can be included in the same model.

Nevertheless, the findings of the current study tentatively suggest that preventing exposure to physical abuse from mothers during childhood, stopping its recurrence, or at the very least tackling the consequences of exposure to this form of abuse, may reduce the likelihood of psychotic disorders developing. Designing and trialing of preventive interventions for psychosis involving avoidance or cessation of physical abuse in childhood are thus required.

## Funding

UK Medical Research Council; the Stanley Medical Research Institute; MRC Population Health Scientist award (G1002366 to H.L.F.).
